# Case Report: Chronic myeloid leukemia in a 13-year-old—a rare pediatric case of extreme hyperleukocytosis in chronic phase

**DOI:** 10.3389/fmed.2026.1809227

**Published:** 2026-04-01

**Authors:** Mohammad Asees, Toleen Abdalhaq, Johnny Amer

**Affiliations:** Department of Allied and Applied Medical Sciences, Faculty of Medicine and Health Sciences, An-Najah National University, Nablus, Palestine

**Keywords:** BCR:ABL1, bone marrow, chronic myeloid leukemia, growth impairment, hyperleukocytosis, Imatinib, pediatric CML, splenomegaly

## Abstract

Pediatric Chronic Myeloid Leukemia (CML), a rare hematologic malignancy, accounts for only 2–3% of cases of leukemia in pediatric patients. The case presented here is of a previously healthy 13-year-old male, who came to the emergency department with increasing fatigue, abdominal distention, and discomfort. On examination, he was pale with significant splenomegaly. His investigation findings included marked leukocytosis (WBC > 600 × 10^9^/L), severe anemia (Hb 5 g/dL), along with thrombocytosis (Platelets > 1,530 × 10^9^/L). The diagnosis was made by peripheral smear and bone marrow biopsy, showing Chronic Phase CML with less than 3% blasts along with intense granulocytic hyperplasia. Molecular studies by PCR identified the BCR:ABL1 fusion transcript. The initial treatment approach emphasized cytoreduction therapy with hydroxyurea, intravenous fluid administration, and preventive medication with allopurinol to protect against the risk of tumor lysis syndrome. After the patient became stabilized, imatinib, a first-line tyrosine kinase inhibitor, was started. The supportive care consisted of transfusion support and gastrointestinal protection. The patient responded quite well to therapy and was therefore discharged with instructions for further follow-up care. As highlighted by this case, the importance of prompt diagnosis, the initiation of cytoreduction therapy, and the use of molecular therapy in treating CML in children cannot be neglected. CML in children is an uncommon but curable form of leukemia.

## Introduction

Chronic myeloid leukemia (CML) is a myeloproliferative neoplasm characterized by uncontrolled proliferation of myeloid cells driven by the BCR:ABL1 fusion oncogene (1). CML is primarily an adult disease, but it can occur rarely in children and adolescents. It comprises approximately 2–3% of childhood leukemias and about 9% of leukemias in adolescents (1, 2). The annual incidence in the pediatric population is very low, on the order of 0.6–1.2 per million children, increasing in older teens (1, 2). Pediatric CML cases often present with more aggressive clinical features compared to adult CML, such as higher leukocyte counts, larger spleen size relative to body mass, and a greater likelihood of advanced-phase disease at diagnosis (1–4). Indeed, an estimated 7–10% of pediatric CML patients present initially in blast crisis (acute leukemia phase), which is higher than in adults (4, 5).

Clinically, children with CML frequently have insidious symptoms or may be asymptomatic; when present, symptoms are often due to hypermetabolism and hyperleukocytosis. These can include fatigue, weight loss, fever or night sweats, bone pain, and left upper quadrant fullness or pain from splenomegaly (1, 2, 4). Serious leukostasis-related complications, such as respiratory distress, visual changes, or priapism are uncommon in chronic phase but may occur when extreme leukocytosis is present (1, 2).

The World Health Organization (WHO) classification traditionally divided CML into chronic, accelerated, and blast phases, but the latest 5th edition (2022) has simplified this by defining only chronic phase and blast phase, with “accelerated phase” features now considered high-risk characteristics within chronic phase (2, 4). Blast phase is defined by ≥20% blasts in blood or marrow or extramedullary blast proliferation, whereas chronic phase CML does not meet those criteria (4).

The advent of tyrosine kinase inhibitors (TKIs) targeting BCR:ABL1 has revolutionized CML treatment. In adults, long-term TKI therapy has transformed CML from a fatal disease into a chronic condition with survival approaching that of the general population (6, 7). In pediatric CML, TKIs have likewise dramatically improved outcomes, obviating the need for upfront stem cell transplantation in most cases (4, 8). However, managing CML in children poses unique challenges, including potential adverse effects of lifelong TKI therapy on growth and development (6, 8). Here, we report a case of CML in a 13-year-old male in chronic phase, and we discuss the diagnostic approach, treatment course, and outcome, with a focused review of the pediatric CML literature.

## Case presentation

A 13-year-old previously healthy male was initially evaluated at a professional lab and diagnosed as a chronic myeloid leukemia (CML) and subsequently referred to An-Najah National University Hospital (NNUH) for further evaluation and management. The patient lives with his family and goes to school. His parents are non-consanguineous. There is no history of congenital disease or blood-related conditions. Most importantly, a week prior to the diagnosis of the child, the patient’s mother was diagnosed with lung cancer. The patient was absent from school due to hospitalization and the start of treatment. However, after the patient began to receive targeted therapy, he was able to return to his regular schooling. Complete blood count showed severe leukocytosis (WBC of 579 × 10^9^/L), anemia (Hb of 6.4 g/dL), and thrombocytosis (Platelet count of 830 × 10^9^/L). Peripheral blood smear taken showed normocytic normochromic anemia with myeloid left shift, indicative of myeloproliferative neoplasm in keeping with chronic-phase CML.

A detailed diagnostic work-up was also conducted after the patient’s admission at NNUH to confirm the patient’s diagnosis. The patient had been experiencing progressive weight loss (11 kg in 2 months), tiredness, generalized weakness, and dyspnea on exertion. On physical examination, he appeared pale with easily fatigable muscles. Abdominal examination revealed that the patient’s spleen was markedly enlarged, extending 29 cm below the left costal margin, crossing the midline, and the liver was enlarged, 4 cm below the right costal margin. Other systems, including cardiopulmonary and neurologic systems, appeared normal with no focal findings.

**Figure 1:**
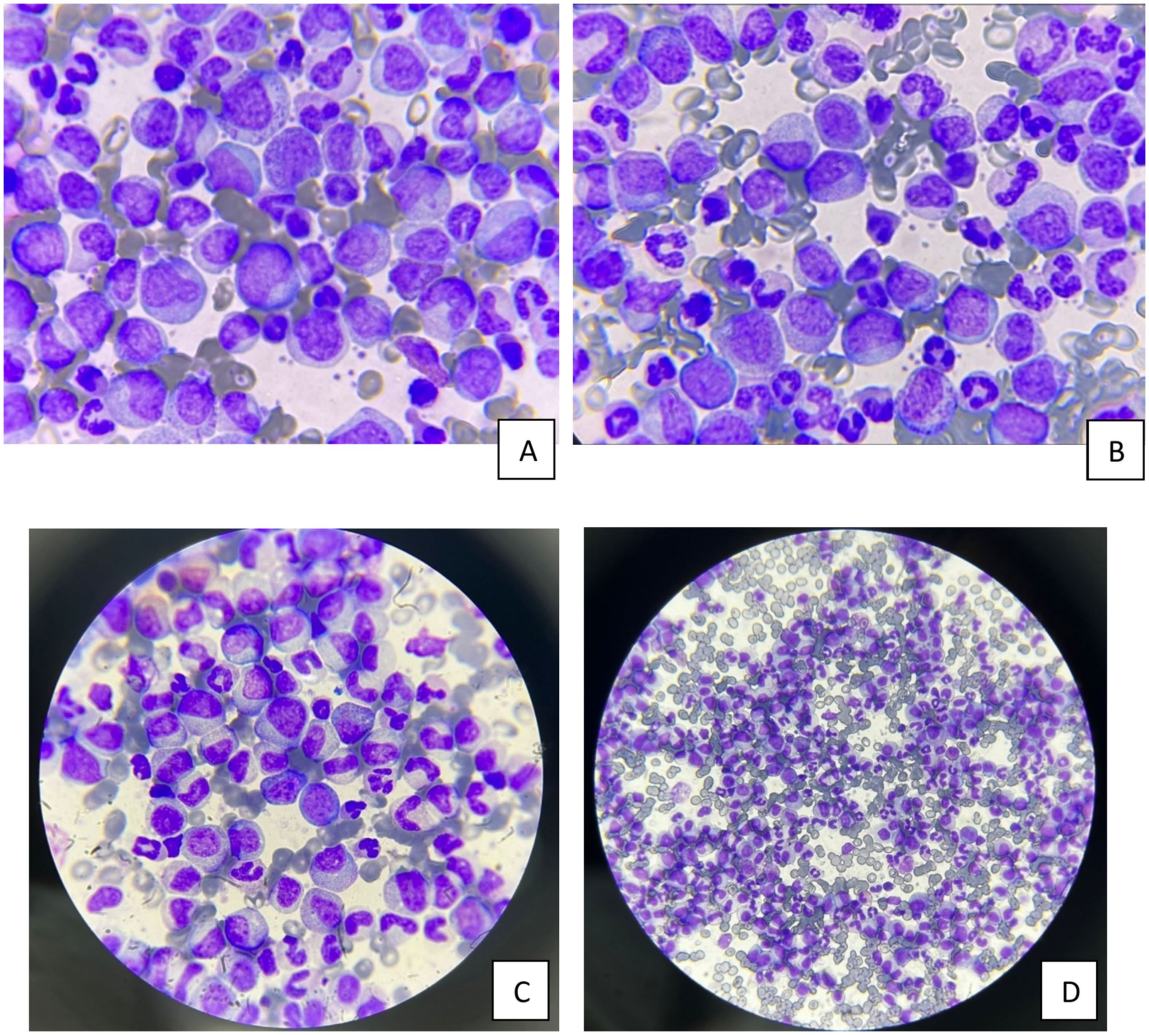
**(A–D)** Peripheral blood smears at diagnosis show marked leukocytosis with granulocytic predominance and left-shifted maturation from myeloblast through mature neutrophils. In addition, occasional blast cells are noted (<5%). These findings correspond with chronic phase chronic myelogenous leukemia.

At NNUH, initial complete blood count showed profound leukocytosis (WBC 607 × 10^9^/L), severe anemia (Hb 5.0 g/dL), and thrombocytosis (platelet counts 1,536 × 10^9^/L), which was consistent with hyperleukocytosis and high-output anemia. Bone marrow aspirate and biopsy showed severe Hypercellularity with granulocytic proliferation and a blast percentage of around 4%, suggestive of chronic phase chronic myeloid leukemia. The flow cytometry analysis of the peripheral blood showed the presence of 2–3% blasts positive for CD34, and the blast percentage as assessed by CD34 immunohistostaining was around 5% of the cellularity in the bone marrow. There was no discordance in the results obtained by the different methods, and all results confirmed the diagnosis of chronic phase chronic myeloid leukemia. BCR:ABL1 testing on peripheral blood was positive. Thus, the diagnosis was confirmed (Figure 1).

Summary of the key hematological values of the patient during hospital stay is provided in Table 1. Also, it should be noted that the CBC test carried out at professional labs one day prior to admission revealed WBC count of 579 × 10^9^/L, together with a Hb level of 6.4 g/dL, and platelets of 830 × 10^9^/L, which highlighted the sharp increase in leukocytosis and the progressive nature of anemia.

**Table 1:** Serial complete blood counts and indices while in-hospital (WBC: white blood cells; Hb: hemoglobin; Plt: platelets; ANC: absolute neutrophil count).

Date	WBC (×10^9^/L)	Hb (g/dL)	Plt (×10^9^/L)	ANC (×10^9^/L)
Outside initial (pre-admission)	579.15	6.4	830	514.82
13/12/2025 (admission day 1)	607.35	5.0	1,536	309.46
14/12/2025 (Day 2)	521.8	5.0	1,225	258.52
15/12/2025 (Day 3)	461.5	5.1	1,246	228
16/12/2025 (Day 4)	480	6.3	1,593	231.2
17/12/2025 (Day 5)	370	6.6	1,390	173
18/12/2025 (Day 6)	331.7	7.0	1,223	149
19/12/2025 (Day 7)	293	7.1	1,430	129.6
20/12/2025 (Day 8)	251	6.9	1,302	116
21/12/2025 (Discharge)	240	7.7	1,447	-

Outside initial refers to a test done at professional lab prior to hospitalization.

On admission, serum biochemistry revealed signs of high cell turnover and anemia-related stress. The lactate dehydrogenase (LDH) was elevated at 1,065 U/L (normal ~100–250), and uric acid was mildly high at 6.8 mg/dL. Kidney function was normal (creatinine 0.84 mg/dL, BUN 11 mg/dL), and electrolytes were within normal ranges except a potassium of 3.7 mmol/L (slightly low, likely from intracellular shift or dilution). Calcium, phosphate, and magnesium levels were normal. Liver function tests were unremarkable (AST 39 U/L, ALT 10 U/L, albumin 4.9 g/dL, total bilirubin 0.43 mg/dL). Inflammatory markers showed a C-reactive protein of 8.1 mg/L (mildly elevated) and erythrocyte sedimentation rate of 1 mm/h (notably low, which can occur in extreme leukocytosis due to altered plasma characteristics). Coagulation screening was within normal limits (PT 14.9 s, aPTT 40.8 s, INR 1.21) (Table 2).

**Table 2:** Timeline of clinical events.

Time	Clinical event
2 months before admission	Progressive weight loss and fatigue
1 day before admission	CBC at outside laboratory showing extreme leukocytosis
Day 1	Admission to An-Najah National University Hospital
Day 2	Abdominal ultrasound showing massive splenomegaly
Day 2	Initiation of cytoreductive therapy with hydroxyurea
Day 3	Bone marrow aspiration and biopsy
Day 3	Confirmation of BCR:ABL1 fusion transcript
Day 3	Initiation of imatinib therapy
Day 9	Clinical stabilization and hospital discharge

### Investigations

On hospital day 2, an abdomen ultrasonography showed massive splenomegaly measuring 29 cm in size involving the pelvis and moderate hepatomegaly measuring 17 cm in size at the mid-clavicular line. Additionally, there was normal echotexture. The two kidneys were normal in size and architecture without evidence of hydronephrosis or calculi. The bladder was underfilled. Moreover, there was a small amount of free fluid in the pelvis. Additionally, qualitative polymerase chain reaction in the peripheral blood revealed BCR:ABL1 fusion gene transcript corresponding to Chronic Myeloid Leukemia.

On Day 3, bone marrow aspiration and biopsy were performed under sedation via the posterior iliac crest. The marrow was markedly hypercellular with pronounced myeloid hyperplasia and fewer than 3% blasts, consistent with chronic-phase CML. Erythropoiesis was suppressed, and megakaryocytes were increased in number, with abnormal morphology, many appearing small and hypolobulated. Reticulin staining showed mild to moderate fibrosis (myelofibrosis grade 2). Immunohistochemical staining for CD61 confirmed the expanded megakaryocytic lineage. There was no increase in lymphoid or plasma cells.

To rule out lymphoid blast crisis and/or mixed phenotype leukemia, flow cytometry analysis was performed using peripheral blood. Blast cell percentage assessment was performed using various diagnostic techniques such as peripheral blood smear, flow cytometry, bone marrow examination, and CD34 immunohistochemistry. Blast cell percentages ranged from 2 to 5%, and this is due to the variability of the techniques and not due to inherent variability. This helps to establish the diagnosis of chronic phase chronic myeloid leukemia.

On hospital day 8, an echocardiogram was performed in anticipation of the use of tyrosine kinase inhibitors. It showed mild dilation of the left atrium and left ventricle with good systolic function (60% ejection fraction), which was attributed to the high output status secondary to anemia. There was no effusion or abnormality.

### Treatment

Upon diagnosis, the priority was to reduce the dangerously high leukocyte count and mitigate the risk of leukostasis and tumor lysis. The patient was started on hydroxyurea, an oral antimetabolite cytoreductive agent, at 1000 mg initially then dose increased to 2000 mg daily. Aggressive hydration was implemented with IV fluids, approximately 1.5–2 times maintenance rate, and allopurinol 300 mg/day was given to prevent hyperuricemia and tumor lysis syndrome. The patient was monitored initially for signs of leukostasis (e.g., neurological changes, respiratory symptoms), but none developed. Over the first 72 h, the WBC count began to decline (Table 1), indicating response to hydroxyurea. The absolute WBC fell from ~607 to 293 × 10^9^/L by the seventh hospital day.

Secondary to the absence of leukostasis and quick response to hydroxyurea therapy, leukapheresis would have been unnecessary in this patient. It also checks in accordance with recommended guidelines in chronic myeloid leukemia.

Initially, the transfusion was withheld to reduce rising viscosity; but subsequent to the decrease in the white blood count, two units of reduced and irradiated packed red blood cells were given for two days. This brought an improvement in the hemoglobin level to 8 g/dL and relieved fatigue and tachycardia. There was no symptom of bleeding, though the platelet count remained high. There was no need for direct treatment for thrombocytosis, as hydroxyurea reduced platelet counts over time.

During cytoreduction, strict monitoring of intake/output and renal function was done. The patient’s electrolytes remained stable and there was no evidence of tumor lysis (normal potassium, phosphate, and calcium; uric acid peaked at 7 mg/dL and then normalized). Allopurinol was discontinued after 4 days once tumor lysis risk had decreased.

Once the diagnosis of CML was confirmed by the positive BCR:ABL1 result, definitive therapy was initiated with imatinib mesylate. On hospital day 3, the patient started Imatinib 400 mg orally once daily, which is the full pediatric dose equivalent to the adult standard (340 mg/m^2^). Imatinib was administered with food and a prophylactic antiemetic, ondansetron 6 mg, 30 min before each dose. The patient tolerated the first doses of imatinib well, with only mild nausea. No acute drop in blood counts occurred in the first few days of TKI therapy, a potential effect as the leukemic clone is suppressed, counts were still high but trending down. Liver enzymes and renal function remained normal on therapy.

Supportive care throughout the hospital course was given, including oral prophylaxis with nystatin and chlorhexidine, aiming to prevent oral candidiasis or mucositis during cytoreduction and early TKI therapy. He started on a proton pump inhibitor, esomeprazole 40 mg daily; for gastric protection, given the combination of medications and perhaps stress ulcer prophylaxis. In addition to ondansetron prior to imatinib, the patient was maintained on a light diet and hydration to minimize gastrointestinal side effects. No broad-spectrum antibiotics were started as there were no signs of infection and the patient’s neutrophil count, although extremely high, comprised largely functional mature cells.

Over the ensuing week on this regimen (hydroxyurea overlapping with the start of imatinib), the patient’s leukocyte count continued to decline. By discharge (Day 9), the WBC had dropped to 240 × 10^9^/L. The platelet count remained elevated (1,447 × 10^9^/L). Both hydroxyurea and imatinib were continued post-discharge, with the plan to taper and discontinue hydroxyurea once adequate hematologic control was achieved with TKI therapy.

The patient was discharged home after 9 days of hospitalization, in clinically stable condition. Discharge medications included imatinib 400 mg daily, hydroxyurea 2 g daily, ondansetron 6 mg as needed, and continued oral hygiene and gastric protection plan. The family received counseling on the importance of adherence to daily TKI therapy and monitoring for side effects. A follow-up visit was scheduled for 3 days post-discharge for repeat blood counts and clinical review.

## Discussion

### Epidemiology and clinical presentation

CML in children and adolescents is a rare entity. It represents only about 2–3% of all leukemia cases in children and roughly 9% of leukemias in the adolescent age group (1, 3).The incidence of pediatric CML is on the order of 1 per million per year, rising in late adolescence (9). There is a slight male predominance reported in some series, though data are limited by the rarity of cases (2). Children with CML typically present in chronic phase (CP), but often with more pronounced clinical features than those seen at diagnosis in adults (4, 9).These features includes higher WBC counts and more massive splenomegaly relative to body size than seen in adult CML (9). One retrospective study found splenomegaly in ~85% and anemia in 80% of children at CML diagnosis (9). Anemia at diagnosis is also commonly observed in pediatric CML and may reflect the degree of bone marrow involvement and disease burden. A recent study by Delehaye et al. reported a high prevalence of anemia at diagnosis in pediatric CML and suggested that hemoglobin levels at presentation may have prognostic implications for disease course and treatment response (10). Constitutional symptoms (fatigue, malaise, weight loss) are also common. Fever and bone pain can occur but are less frequent; in one series only 10% had fevers at presentation (9). Rarely, children may present with complications of hyperleukocytosis such as leukostatic symptoms (e.g., respiratory distress, altered vision, priapism), but this is uncommon in chronic phase because most circulating cells are mature (9). Our patient’s presentation with profound anemia and massive splenomegaly is in keeping with these typical features. He did not experience leukocytosis symptoms despite an extremely high WBC, likely because in CML the leukocytes are predominantly mature granulocytes with lower propensity to cause capillary obstruction compared to blast cells in acute leukemia (9).

It is important to distinguish CML from other causes of leukocytosis in children. The Differential diagnoses for the high neutrophil count with left shift would include a leukemoid reaction. The Leukocyte Alkaline Phosphatase test was historically used as a means of distinguishing these diagnoses, as it tends to be low in CML patients, especially compared with those patients with a leukemoid reaction. However, currently, testing for the presence or absence of BCR:ABL1 is commonly used, as it provides a conclusive result (6). Morphologically, basophilia is a hallmark of CML and is absent in leukemoid reactions (6). Acute leukemias (ALL or AML) can also present with high WBC counts, but they show a predominance of blasts on smear and distinct immunophenotypic profiles (6).

In difficult cases, flow cytometry readily distinguishes CML, which shows a full myeloid maturation pattern with low blast percentage, from acute leukemia. About 5% of pediatric CML cases may present in advanced phases (accelerated or blast phase) (8). Notably, children are somewhat more prone to present in blast crisis compared to adults; approximately 7–10% present in blast phase in pediatric series, often with a lymphoid blast phenotype (9). Our patient was diagnosed in chronic phase; his blast percentage (~2–3%) was well below the 20% threshold for blast crisis, and there were no clonal evolution cytogenetic changes, which bodes well for treatment response.

### Diagnostic approach

Our diagnostic strategy aligned with the international criteria for the diagnosis of chronic myeloid leukemia in the pediatric setting and focused on rapid and precise diagnosis. Once there was suspicion of the disease owing to severe leukocytosis with significant splenomegaly, we immediately performed conventional cytogenetics and BCR:ABL1 quantitative PCR in peripheral blood specimens to start the targeted therapy soon. This dual diagnostic approach is recommended by the National Comprehensive Cancer Network (NCCN) guidelines and international pediatric CML guidelines (2, 4).

Bone marrow aspirations and biopsies, an essential part of pediatric CML evaluation, were done for the evaluation of morphology, fibrosis, and blast percentage. However, according to current international recommendations, bone marrow biopsy is not always mandatory when adequate cytogenetic and molecular testing confirming BCR:ABL1 can be performed using bone marrow aspirate or peripheral blood samples. There was hypercellularity due to granulocytic infiltration with less than 10% blasts, consistent with chronic phase. According to the European LeukemiaNet (ELN) recommendations, chronic phase CML is defined by the absence of criteria for accelerated or blast phase, including blast percentage below 10% in peripheral blood or bone marrow and absence of extramedullary blast proliferation. In our patient, blast percentages ranging between 2 and 5% confirmed the diagnosis of chronic phase CML. These findings are aligned with WHO classification criteria (4). The flow cytometric studies supported these findings by showing 2–3% blasts without lymphoid infiltration.

Additionally, the diagnosis of CML in the chronic phase was supported by the prompt hematologic response to hydroxyurea and the lack of evidence suggestive of either the accelerated or blast phases (such as extramedullary involvement or >20% basophils) (4).

Also, the baseline evaluations of renal and liver function, electrolyte studies, and echocardiogram were normal. These studies are imperative prior to the initiation of tyrosine kinase therapy because of long-term toxicities (4, 8).

The use of various complementary diagnostic tools for the assessment of blasts increased the level of diagnostic accuracy and excluded the advanced phase of the disease because all the tests showed blast percentages to be well below the criteria for the blast phase.

### Management strategies

The initial management for newly diagnosed pediatric chronic myeloid leukemia (CML) patients largely depends on the stage and presenting symptoms. In chronic-phase CML, the prompt start of tyrosine kinase inhibitors (TKIs) is the mainstay of treatment. In cases with extreme hyperleukocytosis (e.g., WBC > 600 × 10^9^/L), cytoreductive therapy with Hydroxyurea is most used and proved to be effective and well-tolerated (2, 4). Leukapheresis is used for symptomatic patients. In pediatric cohorts, one-third of patients with extreme hyperleukocytosis required leukapheresis, while other managed successfully with Hydroxyurea alone, as in our patient (4).

TKI treatment remains the definitive treatment for the chronic phase of CML. Imatinib has remained a standard treatment option for pediatric patients with CML. Dosing in pediatric patients has been through weighing or BSA (260–340 mg/m^2^/day), with many adolescents receiving full standard adult doses (400 mg/day) (4). Clinical studies demonstrate high percentages of complete hematologic and cytogenetic responses among pediatric patients to the similar extents as for adults (2, 4). Second-generation drugs such as dasatinib and nilotinib have found use as treatments for pediatric CML patients, with some situations producing faster responses (2, 8).

Recommendations place a strong emphasis on the principles of treatment according to risk, with selected high-risk patients possibly benefiting from the initial treatment with second-generation TKIs (4). The goals of treatment are to achieve complete cytogenetic response (CcyR) at 12 months and major molecular response (MMR; BCR:ABL < 0.1%) at 18 months. Both are highly predictive of a good long-term outcome (4). We started the patient with imatinib and started seeing some hematologic remission.

Allogeneic hematopoietic stem cell transplantation (HSCT) has now been restricted to those with failure, intolerance, and progression of tyrosine kinase inhibitor therapy. There is no indication of initial transplantation in children with chronic-phase CML (2, 8).

Supportive care is considered integral part in CML management. This includes transfusional support, tumor lysis prophylaxis, and surveillance for toxicities associated with TKI treatment (4). Growth retardation is a unique issue in pediatric patients treated chronically with TKIs. Several series documented a reduction in linear growth in prepubertal patients (6). The patient’s growth is to be carefully monitored. Evaluation by an endocrinologist is done if indicated (6).

Finally, compliance with therapy represents the other important management issue for adolescents. Inadequate compliance with TKI chemotherapy has been identified as the key determinant of failure or relapses of CML. This was adequately highlighted to the patient and family.

### Prognosis

The prognosis of pediatric CML has markedly improved with the introduction of tyrosine kinase inhibitors (TKIs). Before TKIs, survival was limited even with transplantation, especially in the absence of a matched donor. Today, children with chronic-phase CML who respond well to TKIs have long-term survival rates exceeding 90% at 10 years, approaching that of the general population (6, 7).

Although achieving complete molecular remission (undetectable BCR:ABL transcripts) is not essential for a favorable outcome, reaching major molecular remission (MMR) within 12–18 months is strongly associated with excellent prognosis and a very low risk of disease progression (4). In our patient, who was diagnosed in chronic phase and started promptly on imatinib, hematologic remission is expected within 3 months, cytogenetic remission in 6–12 months, and deep molecular remission within 2 years. With continued TKI adherence, the annual risk of progression to blast crisis is less than 1% (4).

The duration of therapy is an evolving topic. In adults, some patients who maintain deep molecular remission (MR) for at least 2 years may attempt treatment-free remission (TFR) under close monitoring, with 40–50% remaining off therapy long term (4). In pediatrics, TFR is not yet standard practice but may be considered in adolescents with sustained deep remission, ideally within research settings (4, 8).

Psychosocially, children with CML can often return to regular school and activities, such as intensive chemotherapy or prolonged hospitalizations are usually not required (8). With daily oral TKI therapy and good support, most patients can maintain a normal lifestyle with a few precautions, such as avoiding contact sports until spleen size normalizes (4).

## Conclusion

This report documents a rare case of Chronic-phase chronic myeloid leukemia (CML) in a 13-year-old male presenting with extreme hyperleukocytosis (600 × 10 (5)/L) and massive splenomegaly. Even though CML is an unusual disease in pediatric patients, it must be included in the differential diagnosis for pediatric cases of severe leukocytosis for which an underlying cause remains unidentified. Laboratory confirmation through BCR:ABL testing is mandatory, with the evaluation of bone marrow still being helpful in determining the disease stage. The positive impact of targeted therapy with imatinib for pediatric CML, after successful cytoreduction with Hydroxyurea, ensures that CML in pediatric patients can be effectively reclassified as a chronic disease that could be made amenable by the use of Tyrosine Kinase Inhibitors (TKIs). Chronic management of CML, in pediatric patients, would thus demand close monitoring of disease response on the molecular level, while taking special care to remain vigilant for possible toxic side effects associated with long-term administration of TKIs.

## Data Availability

The raw data supporting the conclusions of this article will be made available by the authors, without undue reservation.

## Glossary

ALL: acute lymphoblastic leukemia
ALT: alanine aminotransferase
AML: acute myeloid leukemia
ANC: absolute neutrophil count
aPTT: activated partial thromboplastin time
AST: aspartate aminotransferase
BSA: body surface area
BUN: blood urea nitrogen
CcyR: complete cytogenetic response
CML: chronic myeloid leukemia
CP: chronic phase
Hb: hemoglobin
HSCT: hematopoietic stem cell transplantation
INR: international normalized ratio
IV: intravenous
LAP: leukocyte alkaline phosphatase
LDH: lactate dehydrogenase
MMR: major molecular response
MR: molecular remission
NCCN: national comprehensive cancer network
NNUH: An-Najah National University Hospital
PCR: polymerase chain reaction
Plt: platelets
PT: prothrombin time
TFR: treatment-free remission
TKI: tyrosine kinase inhibitor
WBC: white blood cell
WHO: World Health Organization
